# Determinants of uptake of intermittent preventive treatment for malaria with sulfadoxine pyrimethamine in pregnancy: a cross-sectional analytical study in the Sekondi-Takoradi Metropolis of Ghana

**DOI:** 10.1186/s13690-021-00694-1

**Published:** 2021-10-14

**Authors:** Francis Anto, Christabel Ayepah, Elizabeth Awini, Langbong Bimi

**Affiliations:** 1 School of Public Health, University of Ghana, Legon, Accra, Ghana; 2 Western Regional Health Directorate, Public Health Division, Takoradi, Ghana; 3 Dodowa Health Research Centre, Dodowa, GA/R Ghana; 4 Department of Animal Biology and Conservation Science, University of Ghana, Legon, Accra, Ghana

**Keywords:** Intermittent preventive treatment, Sulfadoxine pyrimethamine, Malaria in pregnancy, Secondi Takoradi Metropolis, Antenatal care

## Abstract

**Background:**

Ghana malaria control programme recommends the uptake of five doses of sulfadoxine pyrimethamine (SP) during pregnancy following the review of the World Health Organization recommendations in 2012. The uptake of higher doses of SP since the implementation of the new policy in 2016, has been low across the country. The current study determined factors that can be improved to increase uptake of SP for intermittent preventive treatment of malaria in pregnancy (IPTp-SP).

**Methods:**

A cross-sectional analytical study was carried out among women who had just delivered in selected health facilities in the Sekondi-Takoradi Metropolis of Ghana. Participants were enrolled from the lying-in wards of the study facilities after delivery. Data including time of initiating antenatal care (ANC), number of visits, time of first dose of SP and number of doses were collected. ANC books were also reviewed. Logistic and ordered logistic regression analysis were done to determine respondent factors associated with uptake of IPTp-SP using Stata 15.

**Results:**

Out of the 496 mothers who participated in the study, 370 (74.60%) initiated ANC during the first trimester, 123 (24.80%) during the second, with only three (0.60%) starting during the third trimester. Majority (463/496, 93.35%) made > 4 visits. Uptake of at least one dose of SP was 98.79% (490/496), ≥ 2 doses was 92.75 (460/496), ≥ 3 doses was 80.65% (400/496) and ≥ 4 doses was 40.32% (200/496). Uptake of IPTp 5 was very low (6.65%, 33/490).

A unit increase of one ANC visit was associated with 20% higher odds of receiving 3-4 doses of SP with respect to receiving 1-2 doses (*p* <  0.001). The probability of receiving 5 or more doses of SP with respect to 1-2 doses was 26% higher with a unit increase of one ANC visit.

**Conclusion:**

Uptake of 3-4 doses and ≥ 5 doses of SP were associated with making more ANC visits. Encouraging and motivating expectant mothers to make more ANC visits can improve uptake of ≥5 doses of SP.

**Supplementary Information:**

The online version contains supplementary material available at 10.1186/s13690-021-00694-1.

## Background

Malaria infection during pregnancy is of public health concern as the disease poses significant risk not only to the pregnant woman, but also to her fetus and the newborn child [1]. Most cases of malaria are due to either *Plasmodium falciparum* or *P. vivax*, however almost all malaria deaths are due to *P. falciparum* [2]. In sub-Saharan Africa, *P. falciparum* is the main infecting parasite, and responsible for 99% of all malaria in pregnancy [3, 4]. *Plasmodium falciparum* infection during pregnancy often leads to placental parasitaemia, maternal anaemia, prematurity and eventual low birth weight [5]. Low birth weight is a significant contributor to infant mortality [6]. The impact of the disease during pregnancy depends on the transmission intensity in the given geographical area [7], and the individual’s level of acquired immunity. In high-transmission areas, the levels of acquired immunity tend to be high and so *P. falciparum* infection is usually asymptomatic in pregnancy [8].Due to the high adverse impact of malaria on pregnancy, the WHO recommends the use of long-lasting insecticidal nets (LLINs) and intermittent preventive treatment in pregnancy (IPTp) with sulfadoxine-pyrimethamine (SP), as part of antenatal care services in all areas with moderate to high malaria transmission in Africa. In addition, there should be prompt diagnosis and effective treatment of malaria infections [6]. Intermittent preventive treatment of malaria in pregnancy is a full therapeutic course of antimalarial medicine given to pregnant women at routine antenatal care visits, regardless of whether the recipient is infected with malaria parasites or not [9]. SP is to be given to all pregnant women at each antenatal care (ANC) visit until delivery as directly observed therapy (DOT) by the antenatal care provider. The administration commences early in the second trimester, with doses given at least 1 month apart [10].

IPTp-SP provides protection against malaria based on the number of doses of SP taken [11] and reduces maternal and foetal anaemia, placental parasitaemia and low birth weight [12].

In 2016, the WHO recommended an increase in the number of contacts between health care providers and pregnant women with the aim of increasing IPTp-SP uptake as the intervention has been proven to be an effective means of preventing the adverse consequences of malaria on pregnancy outcomes [13]. Uptake of higher doses of SP is however dependent on individual, community and health facility level factors. These factors include: time of ANC initiation, awareness of the IPTp-SP programme, educational status of the woman [14], number of antenatal care visits, household size, ethnicity, wealth index, place of residence, attitude of health care workers and SP stock-out at health facilities [15, 16].

Currently, the Ghana National Malaria Control Programme (GNMCP) recommends the uptake of at least five doses of SP by the time of delivery following the review of the WHO recommendations in 2012 requiring the uptake of at least three doses during pregnancy. The uptake of five doses of SP since the implementation of the new policy in 2016, has been low across the country [17–19]. According to the GNMCP 2017 annual report, uptake of IPT 5 has been low over the years (1.2, 5.8, 6.7 and 8.9%) for 2014, 2015, 2016 and 2017 respectively [20]. The current study was therefore carried out in selected health facilities in the Sekondi-Takoradi Metropolis, the capital of the Western Region of Ghana, the region that recorded the highest number of malaria in pregnancy cases in 2017, to determine the level of uptake of IPTp-SP. The purpose of the study was to identify recipient related factors that could be improved to increase uptake of five doses SP in Ghana and possibly other endemic areas.

## Methods

### Study design

A cross-sectional analytical study involving women who had just delivered was carried out in selected health facilities in the Sekondi-Takoradi Metropolis, in the Western Region of Ghana over a period of 3 months from March to May, 2019. Participants were enrolled into the study from the lying-in wards of the study facilities after delivery. Primary data including the demographic characteristic were collected directly from the mothers using a pre-tested questionnaire. Their ANC record books were also reviewed and data on obstetric and antenatal care services extracted.

### Study area

The study was carried out in the Sekondi-Takoradi Metropolis located in the south-eastern part of the Western Region of Ghana. The metropolis is bordered to the west by the Ahanta West district and to the east by the Shama district. To the south of the metropolis is the Atlantic Ocean and to the north is the Wassa East district. The metropolis has an estimated population of 555,548 people with a land size of 191.7 km square. It is the most urbanized of all the 22 districts in the Western Region. The metropolis is made up of mainly Ahantas, but all other ethnic groups in Ghana are present in the area [21].

For health care administrative purposes, the metropolis has been divided into four sub-metropolitan areas: Effia Kwesimintsim, Esikado, Sekondi and Takoradi sub-metropolis. There are 76 health facilities in the metropolis: 10 hospitals, three health centres, 54 clinics and nine Community-based Health Planning and Services (CHPS) compounds [22]. Forty-one of the health facilities provide ANC services with 33 of them providing delivery services. Eight of these facilities, Effia Nkwanta Regional Hospital, Takoradi Hospital, Kwesimintsim Hospital, Esikado Hospital, Ghana Ports and Harbours Authority (GPHA) Hospital, UQ Specialist Hospital, Jemima Crentsil Hospital and Our Lady’s Clinic were purposively selected for this study. The selection of these facilities was based on the feasibility of obtaining the required sample size, as higher numbers of deliveries were reported in 2017 and 2018 from these facilities.

### Study population

All mothers who delivered at the selected health facilities within the study period (March to May, 2019) were eligible to be part of this study.

### Sample size estimation and sampling

Using the Cochran formula, *n =* (Z^2^pq)/d^2^ [23], where: *n =* sample size, Z = the z-score that corresponds with 95% confidence interval (1.96), *p =* proportion of pregnant women who received ≥3 doses of IPTp-SP, (46.6%, = 0.466) [24], q = proportion of pregnant women who received < 3 doses of IPTp-SP (1-0.466%, = 0.534), d = margin of error set at 5% (0.05), a sample size of *n* = 382 was estimated. Making room for non-response and missing data of 10%, a minimum sample size of 421 was deemed adequate to detect any differences between mothers who received ≥3 doses of SP and those who could not.

Eight health facilities were purposively selected out of the 33 that provide delivery services in the Sekondi-Takoradi Metropolis. The selection was based on the feasibility of obtaining the required sample size within a reasonably short period of time. Using records on the number of deliveries in the metropolis in 2017 and 2018, two facilities each that recorded higher numbers of deliveries in their respective sub-metropolis were selected. At the health facilities, mothers were enrolled into the study sequentially after delivery and adequate rest but before discharge.

### Inclusion/exclusion criteria

Mothers aged ≥15 years who delivered (both spontaneous and assisted) live babies at the selected health facilities in the Secondi-Takoradi Metropolis during the study period and consented to be part of the study were enrolled into the study. Mothers who were not very well after delivery or declined consent were excluded from the study.

### Data collection procedure and tool

Data were collected directly from the mothers whilst on the lying-in wards after delivery and adequate rest; using an interviewer-administered questionnaire. The questionnaire had two main sections: a) socio-demographic characteristics and insecticide treated net (ITN)/LLIN use; b) medical and obstetric history of the mother. Data on socio-demographic characteristics of the mother included age, marital status, educational level and occupation. The ANC record books were also reviewed and data on her medical and obstetric history extracted. These data included the following parameters: 1. gestational age at first ANC, 2. total number of ANC visits before delivery, 3. gestational age at taking the first dose of SP, 4. number of SP doses taken before delivery, and any malaria infection during the most recent pregnancy. Data on haemoglobin readings at ANC initiation and time of delivery were also extracted from the ANC record books.

### Data processing and analysis

Data were entered using Excel version 2013, cleaned and imported to Stata version 15 for analysis. The number of doses of SP taken was categorised into < 3 and ≥ 3 doses; and < 5 and ≥ 5. The socio-demographic and ANC characteristics of the mothers (age, number of ANC visits and time of initiation) were also grouped. Age was put into five groups at intervals of 5 years as shown in Table 1. Bivariate analysis was done using Pearson chi square tests to assess association between uptake of ≥3 doses of SP and timing of ANC visit and other independent categorical variables. Factors with *p*-value < 0.05 at 95% CI were considered statistically significant. For the binary categories of uptake of < 3 doses of SP and ≥ 3 doses of SP, logistic regression analysis reporting odds ratios was carried out to control for other factors such as maternal age, number of ANC visits and maternal educational level and to estimate the strength of association. With three categories of IPT uptake, 1-2 doses of SP, 3-4 doses of SP and ≥ 5 doses of SP, ordered logistic regression analysis was done to identify factors associated with SP uptake. Proportion odds model (POM) was employed for the analysis after testing for proportionality odds assumption. The test statistics indicate that both proportionality and parallel regression assumption have been met. The test statistics are indicated in the results for the model (Table 4).

**Table 1:** Background characteristics of study mothers, Sekondi-Takoradi Metropolis, 2019

Characteristics	Frequency (***n*** = 496)	Percentage (%)
**Age (yrs)**		
15-19	17	3.43
20-24	70	14.11
25-29	146	29.44
30-34	151	30.44
35 and above	112	22.58
**Marital status**		
Married	461	92.94
Single	35	7.06
**Educational Level**		
No formal education	1	0.20
Basic	197	39.72
Secondary	219	44.15
Tertiary	79	15.93
**Employment status**		
Government/private sector	138	27.82
Self-employed	285	57.46
Unemployed	73	14.72
**Number of children alive**		
1-2	289	58.27
3-4	191	38.51
5-6	16	3.23
**Trimester of pregnancy at first ANC**^**a**^		
First trimester	370	74.60
Second trimester	123	24.80
Third trimester	3	0.60
**Number of ANC visits**		
1-4	33	6.65
5-7	230	46.37
≥ 8	233	46.98
**Use of ITN the previous night (declared)**		
Used	250	50.40
Did not use	246	49.60

^a^First trimester = 4 -12 weeks of gestation; Second trimester = 13-24 weeks of gestation; third trimester = ≥25 weeks of gestation

### Quality control

Two research assistants were recruited for each health facility. These assistants were either trained midwives or enrolled nurses. They were trained over a period of 2 days on all aspects of the study including, how to conduct the interviews, complete the questionnaire and extract relevant data from the ANC record books of the mothers. The questionnaire was pretested at the Discove District Hospital which is in the adjacent Ahanta West district with similar characteristics in terms of ANC service delivery. All questionnaires were checked in the field for completeness on daily basis.

## Results

### ANC initiation and anaemia status of study participants

**Fig. 1:**
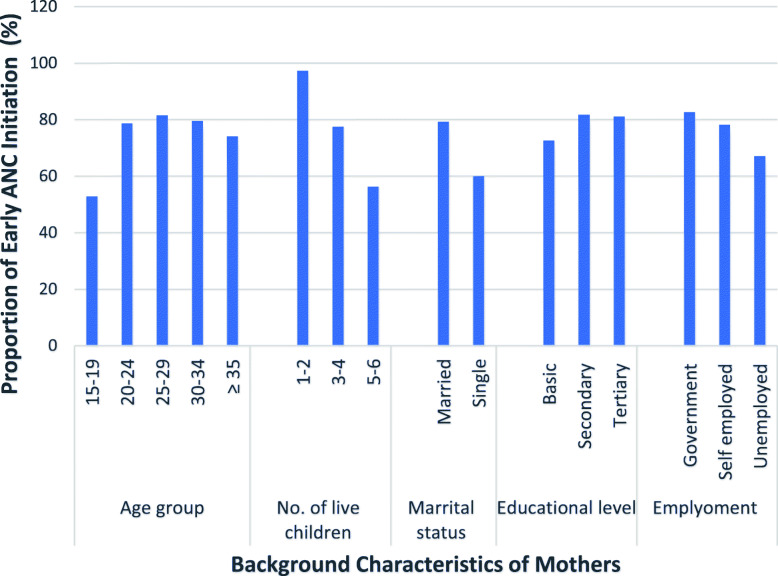
Characteristics of Mothers who Initiated ANC Early (first trimester), Sekondi-Takoradi Metropolis, 2019

Majority of the mothers who initiated ANC early (first trimester) were aged 20 years and above, married and employed (in the government sector or self-employed) (Fig. 1). Two hundred and thirty-six (47.58%) of the women had anaemia (Hb < 11.00 g/dl) at the time of ANC initiation. Half of the women (225/446) whose haemoglobin levels were checked at term was found to be anaemic (Table 2). Among them, 33.33% were not anaemic at the time of initiating ANC. The anaemia status of the women at term was not related to the number of doses of SP taken.

**Table 2:** Relationship between ANC characteristics, socio-demographic characteristics and IPTp-SP uptake among recently delivered women, Sekondi-Takoradi Metropolis, 2019

Variables	Frequency	IPTp-SP uptake (%)		χ^**2**^	p-value
	(***n =*** 496)	< 3 doses	≥ 3 doses		
**Trimester of pregnancy at first ANC**					
First trimester	386	15.54	84.46	16.194	< 0.001
Second + third trimester	110	32.73	67.27		
**Number of ANC visits**					
1-3	33	30.30	69.70	27.77	< 0.001
4-7	230	27.83	72.17		
**≥** 8	233	9.44	90.56		
**Number of children**					
1-2	289	18.69	81.31	3.490	0.175
3-4	191	18.85	81.15		
5-6	16	37.50	62.50		
**Marital Status**					
Married	461	18.44	81.56	3.517	0.61
Single	35	31.43	68.57		
**Educational Level**					
Basic + no formal education	198	24.24	75.76	5.372	0.068
Secondary education	219	16.89	83.11		
Tertiary education	79	13.92	86.08		
**Age group**					
15-19	17	41.18	58.82	8.356	0.187
20-24	70	15.71	84.29		
25-29	146	17.81	82.19		
30-34	151	20.53	79.47		
35 and above	112	18.75	81.25		
**Malaria infection during pregnancy**					
Had infection	76	44.74	55.26	37.045	< 0.001
Had no infection	420	14.76	85.24		
**Anaemia at term**					
Was anaemic	225	18.67	81.33	0.001	0.974
Was not anaemic	221	18.10	81.90		

*IPTp-SP* Intermittent preventive treatment in pregnancy with sulphadoxine pyrimethamine, *n* = number of respondents.

### Background characteristics of study participants

A total of 496 mothers aged 15-49 years (median: 30, IQR: 26-34) participated in the study. A few of the mothers (17/496, 3.43%) were aged < 20 years. Most of them were married (92.94%) and had at least basic level education (99.80%). Majority of the mothers (423/496, 85.28%) were gainfully employed and had one or two children (58.27%). Three hundred and seventy (74.60%) of the mothers initiated ANC during the first trimester (early initiation), 123 (24.80%) booked during the second, with only three (0.60%) starting during the third trimester. Majority of the mothers (93.35%) made > 4 ANC visits before delivery and half of the study participants (50.40%) reported sleeping under an ITN the night before the study (Table 1).

### Uptake of IPTp-SP

Uptake of IPTp-SP was found to be high; with 98.79% (490/496) of the women taking at least one dose. The proportion of women who took two - five doses of SP was as follows: 2 doses = 12.1%, 3 doses = 40.32%, 4 doses = 33.67% and 5 doses = 6.65%. Six of the women (1.21%) did not take any dose of SP. These six women were glucose-6-phosphate dehydrogenase (G6PD) deficient and so were not eligible to take SP. Uptake of ≥2 doses was 92.75% (460/496), ≥ 3 doses was 80.65% (400/496), ≥ 4 doses was 40.32% (200/496and uptake of 5 doses was 6.65% (33/490).

### Relationship between socio-demographic characteristics, ANC visits and IPTp-SP uptake

Bivariable analysis (Table 2) revealed that uptake of ≥3 doses of SP was significantly higher among mothers who initiated ANC during the first trimester (early), compared with those who started during the second or third trimesters (late) (χ^2^ = 25.136, *p* < 0.001). None of the three women who initiated ANC during the third trimester was able to take three doses of SP. The number of ANC visits made also significantly influenced the ability of the woman to receive ≥3 doses of SP. Most (90.56%) of the women who made ≥8 visits were able to take ≥3 doses, whilst 72.17% of those who made 4-7 visits took ≥3 doses, with 69.70% of those who made three visits taking three doses. These differences are statistically significant (χ^2^ = 27.777, *p* < 0.001).

The number of children that a woman had and her marital status did not influence her uptake of IPTp-SP. Similarly, her educational level and age had no influence on her uptake of the drug. A higher proportion (85.24%) of participants who took ≥3 doses of SP compared with those who received < 3 doses (14.76%) was without malaria infection throughout the period of the pregnancy (χ^2^ = 37.045, *p* < 0.001). Uptake of ≥3 doses of SP however, did not influence haemoglobin levels at the time of delivery (Table 2).

Logistic regression analysis controlling for possible confounders revealed that uptake of ≥3 doses of SP was dependent mainly on early initiation of ANC. Uptake of ≥3 doses of SP was significantly higher among mothers who initiated ANC during the first trimester compared with those who started during the second or third trimesters in both the unadjusted and adjusted analysis (*p* < 0.001). Though, uptake was about two times higher among mothers who made ≥4 visits compared with those who made < 4 visits in the unadjusted analysis, it was not statistically significant (*p* = 0.104) (Table 3) and this effect further reduced in the adjusted analysis. Similarly, though uptake of SP was significantly higher in the mothers aged between 20 and 24 years compared with those 15-19 years in the unadjusted, it was not in the adjusted analysis.

**Table 3:** Logistic regression analysis to determine association between ANC characteristics of respondent and uptake of ≥3 doses of IPTp-SP

Characteristics	COR (95%CI)	***p***-value	AOR (95%CI)	***p-***value
***Trimester at first ANC visit***				
First trimester	1.00		1.00	
Second + third trimesters	0.38 (0.23, 0.61)	< 0.001	0.40 (0.24, 0.67)	< 0.001
***Number of ANC visits***				
< 4	1.00		1.00	
≥ 4	1.91 (0.88,4.15)	0.104	1.24 (0.52, 2.93)	0.630
***Educational level***				
Basic	1.00		1.00	
Secondary	1.57(0.97,2.54)	0.064	1.59 (0.93,2.73)	0.090
Tertiary	1.98 (0.97, 4.04)	0.062	1.91 (0.89, .07)	0.095
**Age group**				
15-19	1.00		1.00	
20-24	3.93 (1.15, 13.48)	0.029	2.88 (0.81, 10.26)	0.103
25-29	2.88 (0.96, 8.65)	0.059	1.62 (0.50, 5.25)	0.418
30-34	2.40 (0.81., 7.13)	0.115	1.42 (0.45, 4.50)	0.549
35 and above	2.73 (0.89,8.38)	0.079	1.89 (0.59, 6.07)	0.285

*COR* crude odds ratio, *AOR* adjusted odds ratio, *95% CI* 95% confidence interval.

### Ordered logistic regression analysis to determine factors associated with meeting WHO’s recommendation of ≥3 and Ghana’s ≥ 5 doses of SP

Table 4 presents results from the ordered logistic regression. The satisfaction of the proportional odds assumptions (*p* = 0.452) implies that the relationship between each pair of outcome category (categories of SP doses) are the same. There is thus only one set of coefficients or odds ratios that describes the relationship between the covariables and SP uptake though SP uptake has three categories.

**Table 4:** Ordered Logistic Regression (POM) to examine the relationship between uptake of IPTp doses and respondent factors

Co-variable	Regression coefficient	Standard error	***p***-value	OR (95% CI)	Single score test (***p***-value)
**Gestational age at first ANC visit (First trimester as reference)**					
Second + third trimesters	−0.69	0.25	0.006	0.50 (0.31,0.82)	0.178
**Educational level of mothers (Basic level as reference)**					
Secondary	0.50	0.24	0.041	1.65 (1.02,2.66)	0.531
Tertiary	0.55		0.099	1.74 (0.90,3.37)	
**Maternal age (15-19 years as reference)**					
20-24 years	1.06	0.60	0.077	2.90 (0.89,9.45)	0.680
25-29 years	0.39	0.57	0.502	1.47 (0.48,4.53)	
30-34 years	0.33	0.57	0.501	1.47 (0.48, 4.47)	
35+ years	0.60	0.57	0.296	1.81 (0.59, 5.539)	
**Number of ANC visits (One visit as reference)**					
Unit increase of one visit	0.15	0.04	< 0.001	1.16 (1.07,1.26)	0.149
**Use of ITN (Yes as reference)**					
No	−0.05	0.23	0.822	0.95 (0.61, 1.49)	0.615

**Score test for proportional odds assumption for the model:** Chi-square = 4.52, df = 5, *p-*value = 0.477.

**Brant test of Parallel regression assumption:** Chi-square = 4.82, df = 5, *p*-value = 0.438.

*OR* Odds ratio, *95% CI* 95% confidence interval.

The time of ANC initiation was statistically significant among the factors that determined the uptake of SP doses by the expectant mothers. Those who initiated ANC in the first trimester were more likely than those who initiated ANC in the second and third trimesters to receive more doses of SP. The odds of a mother who initiated ANC in the second and third trimesters receiving more doses of SP was about 50% of that of those who initiated ANC in the first trimester (*p* = 0.006).

The number of ANC visits during pregnancy was also statistically significant among factors that determined the uptake of more doses of SP. A unit increase of one ANC visit was associated with about 16% higher odds of receiving more doses of SP (*p* < 0.001). Thus, the probability of receiving more doses of SP is about 16% higher with a unit increase of one ANC visit.

The level of education of the mother was statistically significant among factors that determined the uptake of more doses of SP. Mothers with secondary education were about 65% more likely than those with basic education to receive more doses of SP. Though mothers with tertiary education were about 74% more likely to receive more doses of SP, the effect was not statistically significant. The use of insecticide treated bednet (ITN) by mothers was not statistically associated with IPTp uptake. However, mothers who did not use ITN were 5% less likely to receive more doses of SP.

## Discussion

A cross-sectional analytical study was carried out among mothers who had just delivered live babies at selected health facilities in the Secondi-Takoradi Metropolis of Ghana. The study identified individual level factors associated with uptake of three and five doses of IPTp-SP as recommended by WHO and Ghana respectively. The study established that early initiation of ANC, making more ANC visits and having secondary level education were the main factors associated with uptake of higher doses of SP.

The current study reports a high proportion (74.60%) of pregnant women initiating ANC during the first trimester (early). This level of early initiation of ANC is much higher than was found in our earlier studies (ranging from 30 to 46%), which found most of the women starting ANC during the second trimester [17, 18, 24]. A recent report by Vandy and colleagues also from Ghana, found less than 50% of the women initiating ANC during the first trimester [19]. Even much lower levels of early initiation of ANC have been reported from some other malaria endemic areas in Eastern (39.4%) [25, 26] and Central (2.2%) Africa [27].

Early initiation of ANC in the current study, was associated with being aged 20 years and older, married and working in the government sector. Other studies have also reported association between early initiation of ANC and individual and service delivery factors, including the educational level of the woman and the number of living children [28, 29]. Also, household wealth [30], charging of fees during booking for ANC and whether the particular pregnancy was planned or not [26], have been associated with early initiation of ANC.

The high level of early initiation of ANC found in the current study may be due to the fact that a high proportion of the women have secondary and higher level education (60%) [31], and possibly having adequate knowledge on IPTp-SP [32]. Most of these women (85.28%) were also gainfully employed [33]; thus, empowering them to make decisions on issues affecting their health. All of them have also delivered before, hence they would have participated in health education sessions organized by nurses and midwives at ANC on the need for early booking. Our study area is also a metropolitan capital, and so the issue of distance in terms of geographical access will not be a hindrance to ANC visits.

This high level of early initiation of ANC enabled the women to make more visits with over 90% of them making more than the minimum recommended number of four visits by WHO, and 47% being able to make the required eight or more visits [33]. Consequently, a high proportion of the women (over 80%) were able to receive ≥3 doses of SP as recommended by the WHO. Though, the study by Vandy and colleagues in the Volta Region of Ghana reported a lower level of early initiation of ANC, a high proportion (82.1%) of the women were able to meet the WHO recommendation of ≥3 doses of SP. Also, many more women (17.1%), relative to our current 6.65%, received Ghana’s five dose coverage recommendation. Obviously, there should be other factors, possibly the number of ANC visits made that should be playing a more critical role in the uptake of five or more doses of SP besides early initiation of ANC.

Thus, the early initiation of ANC recorded in the current study, did not translate into meeting Ghana’s target of five 5 doses of SP. Uptake of five doses of SP was very low with only 6.65% of the women being able to meet the target. This level of uptake, was much lower than earlier reports of 14.5% [17] and 16.0% [18] from other parts of the country. For the few women who were able to meet the five-dose target, more than half (56.58%) of them had to make ≥8 ANC visits.

The ordered logistic regression analysis revealed that the number of ANC visits during pregnancy was significantly associated with the uptake of 5 or more doses of SP [19] as against 1-2 doses. Thus, if a pregnant woman initiates ANC during the second trimester and receives the first dose of SP at week 16 gestational age and continuous to meet all regular ANC appointments, till delivery, she should be able to make the minimum number of visits (five) to enable her take five doses of SP before delivery. In our study area, pregnant women are scheduled for ANC visits at 4 weeks intervals at early pregnancy to 32 weeks gestation. Visits are scheduled more frequently (2 weeks intervals) from 32 weeks to 36 weeks, and then at weekly intervals from 36 weeks to delivery. Such scheduled visits enabled many of the women to made ≥8 ANC visits. This can be exploited to help fill-in any missed SP doses to help meet the targeted five dose regimen, but ensuring that there is the 4 weeks spacing between doses.

Generally, women who initiate ANC early are much more likely to receive the WHO recommended services than those who start late [15, 25]. According to Agha and Tappis, however, uptake of these services is independent of a range of socio-economic and demographic factors and independent of the number of ANC visits made during pregnancy [30]. Thus, some other factors possibly service-related factors, (which were not investigated in the current study), might have contributed to the inability of most of the women to take five doses of SP. Earlier studies have implicated some service related factors including, unavailability of SP at the time of ANC [34] and insufficient time for proper antenatal care counselling by health workers as driving factors for inadequate IPTp delivery [14, 24]. By this, if the service-related factors (e.g. availability of SP) are adequately addressed, and midwives spend adequate time to educate mothers on the importance of high doses of SP, increased ANC attendance could enable the pregnant woman meet the required 5-dose target. Community involvement, especially opinion leaders and men in general may also help improve uptake of SP. In the current study, six women did not take any dose of SP, as they were G6PD deficient.

The study had some limitations as it was focused on identifying individual level factors that could be addressed to help achieve the IPTp-SP target of three and five doses set by WHO and the Ghana malaria control programme respectively. Service and community factors such as stock-out, staff strength, time for education/counselling, travel distance, and financial cost to the women, that could explain some of the findings of this study were not considered. The health facilities were purposively selected based on the fact that they usually report the highest number of antenatal attendants and deliveries in the metropolis. This mode of selection may introduce some biases that should be taken into consideration when interpreting the findings of the study. These limitations notwithstanding, valuable information has been provided to inform programme implementers that, the women are initiating ANC early and some are making eight and more visits and yet are not getting the required number of SP doses.

## Conclusions

The current study has established that making more ANC visits at the appropriate times can help improve uptake of IPTp-SP to meet the three-dose and the five-dose targets set by the WHO and Ghana malaria control programme respectively. Innovative service delivery strategies including behaviour change communication that will encourage and motivate mothers to make more ANC visits may improve coverage of the IPTp-SP policy in Ghana and other malaria endemic areas.

## Supplementary Information

**Additional file 1.** Anto et al data on IPTp.

## Data Availability

All data generated during the study are included in the current published work and its supplementary information file (Additional file 1).

## Abbreviations

ANC: Antenatal Care
CHPS: Community-based Health Planning and Services
GPHA: Ghana Ports and Harbours Authority
IPT: Intermittent Preventive Treatment
